# Associations Between Different Subtypes of Adverse Childhood Experiences with Reproductive Mood Disorders—a Scoping Review

**DOI:** 10.1007/s11920-026-01714-z

**Published:** 2026-08-29

**Authors:** Christian Eric Deuter, Eugenia Kulakova, Katja Wingenfeld, Linn Kristina Kuehl

**Affiliations:** 1 Charité – Universitätsmedizin Berlin, Klinik Für Psychiatrie Und Psychotherapie, Corporate Member of Freie Universität Berlin, Humboldt-Universität Zu Berlin, and Berlin Institute of Health, Campus Benjamin Franklin, Berlin, Germany; 2 Faculty of Natural Sciences, Clinical Psychology and Psychotherapy, Department of Psychology, MSB Medical School Berlin, Berlin, Germany; 3 German Center for Mental Health (DZPG), Partner Site Berlin, Germany

**Keywords:** Adverse childhood experiences, Reproductive mood disorder, Premenstrual syndrome, Premenstrual dysphoric disorder, Postpartum depression, Perimenopausal depression, Sexual hormones, Estradiol, Progesterone

## Abstract

**Purpose of Review:**

Growing evidence suggests that adverse childhood experiences (ACE) increase the risk for reproductive mood disorders (RMD), i.e. premenstrual syndrome, premenstrual dysphoric disorder, postpartum and perimenopausal depression, and might be associated with symptom severity in these disorders. However, evidence for the specific impact of ACE subtypes remains scarce. This review investigates whether certain ACE subtypes are more prevalent among women with RMDs, whether women exposed to ACE have a higher risk to develop such disorders and if the degree of exposure is associated with the symptom severity.

**Recent Findings:**

We conducted a literature search across electronic data bases and reviewed a final selection of 16 studies that differentiated between ACE subtypes.

**Results:**

The findings indicate that emotional abuse tends to be the subtype most clearly associated with these disorders. While emotional abuse was consistently linked to adverse outcomes, the complexities of ACE co-occurrence and the potential differing impacts of other subtypes such as different forms of abuse and neglect are underscored. The review highlights the variability in study populations, methodologies, and assessment tools.

**Summary:**

The interplay between hormonal fluctuations and emotional dysregulation is discussed as potential mediators in the development of RMDs, suggesting that emotional abuse may exacerbate sensitivity to hormonal changes. Future research is called for to clarify subtype-specific mechanisms, age-related effects, and genetic predispositions to hormonal sensitivity following ACE. Overall, this article contributes to the understanding of how ACE subtypes intersect with the etiology of RMDs in women, emphasizing the need for tailored approaches in research and clinical interventions.

## Introduction

The influence of sex and gender on mental disorders has been increasingly recognized in recent years [25]. Some mental disorders do not affect men and women^1^ to the same degree, i.e. women are approximately twice as likely to develop a depression or anxiety disorder in the course of their lives [7, 35]. Interestingly, this female sex bias in depression and anxiety manifests itself only during the reproductive period, i.e. from puberty to menopause, whereas before and after these biological life transitions that mark the onset and end of ovarian hormone fluctuations, prevalence does not differ significantly from men [22]. In addition to higher prevalence of these two most common mental health diagnoses, several disorders exclusively affect women because they are associated with fluctuations of estradiol and progesterone across the menstrual cycle, during pregnancy and after childbirth or related to menopause transition [40]. Premenstrual syndrome (PMS) and, in its more severe form, premenstrual dysphoric disorder (PMDD), postpartum depression (PPD) and perimenopausal depression have been conceptualized as ‘RMDs’, a term we will use in this review [49, 81, 82]. They are characterized by affective symptoms (i.e. increased negative affect and decreased positive affect), affective lability and functional impairment (i.e. clinically significant distress or disability in social, occupational, or other important activities) that occur during specific reproductive states. While they each have unique characteristics, they share an increased sensitivity to hormonal fluctuations as a common etiological factor [5, 85]. In the following, we will briefly describe these RMDs and their endocrine foundations.

The monthly menstrual cycle can be divided into four distinct phases, which are defined by ovulation and menses: the early follicular, mid-cycle/ovulatory, luteal, and premenstrual/late luteal phase, however, many studies simply distinguish the pre-ovulatory follicular phase and the subsequent, post-ovulatory luteal phase [83]. The follicular phase begins on the first day of menstruation, lasts an average of 13–14 days until ovulation and involves the maturation of follicles in the ovaries. The luteal phase is the second half of the menstrual cycle; it begins after ovulation and ends the day before the next menstrual period. The different phases of the cycle are defined by characteristically fluctuating levels of the two sex hormones estradiol and progesterone, which in turn have an influence on emotional, motivational and cognitive processes. These monthly fluctuations are regularly experienced by all naturally cycling (i.e. in the absence of hormonal contraception) females of reproductive age. Effects on psychological processes and behavior are common and not necessarily pathological; up to 90% of women are impacted by some form of usually mild impairments [56]. However, some women respond stronger to the fluctuating levels of these hormones and develop clinically relevant symptoms. Women affected by the PMS/PMDD suffer from emotional sensitivity, anxiety, irritability and depressed mood at the end of the menstrual cycle during the late luteal phase. Premenstrual symptoms are common, with about a third of all women of reproductive age suffering from PMS, while estimates for PMDD range from 2.4% [39] to as high as 17.6% in young adult women [18]. While such fluctuations affect women on a regular basis throughout the reproductive period, there are certain time windows in a woman's life that are characterized by pronounced transitions in the production of sex hormones. Pregnancy requires major adaptations and the reorganization of ongoing endocrine and physiological processes to accommodate for the maturation of the embryo. Estradiol and progesterone levels increase continuously after conception, and decline sharply after parturition. These changes are by magnitudes more pronounced than the cycle-related fluctuations and the withdrawal after birth is a contributing factor for postpartum depression (PPD). PPD is a severe, longer-lasting depressive disorder with an onset in the first year after childbirth that can persist for several years [97]. With a prevalence of 15–20%, PPD is the most common complication of childbirth [8, 97]. Besides the postpartum period, the pre- and perimenopause is a further period characterized by significant reduction in sex hormone levels in a woman's life. The transitional phase of the perimenopause is characterized by strong fluctuations of these two hormones, which terminate postmenopausal with overall low levels. Menopause, defined as the last occurrence of menses, marks the end of the fertile time window and is preceded and followed by various physiological changes as well [78]. At the endocrine level, the production of the gonadal hormones declines significantly. As with the hormonal fluctuations of the menstrual cycle and postpartum, mood swings and irritability are common during this period and as such are not necessarily pathological. However, many women also experience clinically relevant symptoms, with depression prevalence in perimenopausal and postmenopausal women of approximately 35% [43, 100].

Yet, while these disorders are linked to sex hormones fluctuations, these fluctuations are part of the biological functioning of the female body, and only a small percentage of women develop clinically relevant symptoms as a result [2, 12, 81, 85, 99]. Even if the exact mechanisms are not yet fully understood, the reason why some women suffer more and develop clinically relevant symptoms is likely related to increased sensitivity to these fluctuations, rather than hormonal withdrawal or the magnitude of the fluctuations or hormone levels per se [52, 63]. Among other factors, recent research points towards adverse childhood experiences (ACE) to play an important role in the developmental calibration of the gonadal system and as a risk factor for this increased sensitivity to hormonal fluctuations [93]. The term ACE refers to negative, stressful or traumatic events during childhood and adolescence and has significant overlap with related concepts such as Early Life Stress, Childhood Adversity or Childhood Trauma, with these terms often being used interchangeably [73]. Due to its widespread and established use, we will use the term ACE in this article. The detrimental effects of ACE on mental health have been repeatedly demonstrated and are widely reviewed [1, 87, 94]. We do not want to imply that the gonadal hormone system is the primary mechanism of ACE effects, as ACEs affect various developmental processes and different biological pathways are discussed for ACE effects on mood disorders [38, 95]. However, it is noteworthy that ACE effects on different domains are sex-specific, e.g. the influences of ACE exposure on biologically driven developmental processes such as pubertal timing are more evident in girls [50]. The dysregulation of the gonadal hormonal system and increased sensitivity to hormonal fluctuations provides a promising model for sex-differential effects of traumatic stress, might explain the greater susceptibility of ACE-affected women to mood disorders, and in general could provide a mechanistic link between ACE and RMDs [74, 75, 79, 93, 98]. With this review we investigate if individual ACE subtypes are differentially associated with RMDs.

ACE is multifaceted, and the widely used childhood trauma questionnaire (CTQ) differentiates between emotional and physical neglect on the one hand, and emotional, physical and sexual abuse on the other [9]. Other inventories such as the Adverse Childhood Experiences Questionnaire (ACE-Q) include the dimension of household dysfunction (mother treated violently (domestic violence exposure), household substance abuse, household mental illness, parental separation or divorce or an incarcerated household member in the family) or further categories [14]. These dimensions are often aggregated into a global score to investigate the detrimental effects of ACE. However, the question of specific effects of different subtypes of ACE has raised increasing interest in recent years and is the subject of controversial debate. The unspecific investigation of ACE, what Kim et al. (2025) consider as the “lumping approach”, does not differentiate subtypes of adversity but groups them together as one general factor. In contrast, the “specificity approach” refers to conceptualizing and analyzing individual types of adverse childhood experiences separately, rather than combining them into a single cumulative ACE score [47]. According to this approach, each individual adversity is regarded as unique in its consequences and developmental trajectories. Somewhat in between are dimensional approaches to ACE, with the classification of ACEs by McLaughlin et al. [57, 58] in either the threat/harm dimension on the one hand, and the deprivation/loss dimension on the other, as the most prominent examples. Studies that differentiated between subtypes found that they affect various traits, psychological functioning and mental health outcomes and, in the case of multiple adversities, may synergistically interact and potentiate these effects [14, 24, 33, 101]. While sexual and physical abuse as serious and obvious forms of traumatic stress have long been the focus of empirical studies, the detrimental effects of more “silent” forms of maltreatment [41] such as verbal and emotional abuse and neglect have become increasingly apparent [29, 64, 72]. In fact, recent findings indicate that emotional abuse and neglect might have as harmful and damaging developmental consequences on mental health outcomes as other severe forms of maltreatment – potentially due to their detrimental effects on self-perception – with emotional abuse posing the highest risk for developing a lifetime depression [19, 28, 55, 91]. Yet, as [51] point out with respect to PMDD, whether these findings are generalizable to psychiatric conditions with a pronounced hormonal component is unknown. In contrast, sexual and physical abuse could have a stronger influence on reproductive disorders with an assumed dysregulation of the sex hormone system. These forms of maltreatment appear to have a stronger influence on biologically mediated developmental processes such as accelerated biological aging and pubertal onset [66, 92]. Furthermore, it has been described that the effects of gonadal hormones on sexual function in women are moderated by exposure to specifically sexual traumatic experiences [79].

With this review, we want to provide an overview of studies investigating the specific effects of different forms or subtypes of ACE on RMDs. We want to address the question of whether these disorders are predominantly affected by emotional abuse and neglect or, due to their strong biological component, rather by sexual or physical abuse or physical neglect. We will focus on RMDs for which there is a described link to fluctuations in sex hormones and for which there is at least one article showing an influence of ACE broken down into subtypes. Accordingly, we will concentrate on menstrually related mood disorder and perimenopausal depression. Another disorder that fulfills the above criteria is postpartum depression. However, since there are already excellent review articles on this matter and only recently a comprehensive review of relevant findings was published [17, 26], we summarize the findings of this recent review with regard to subtype-specific effects.

## Methods

**Fig. 1:**
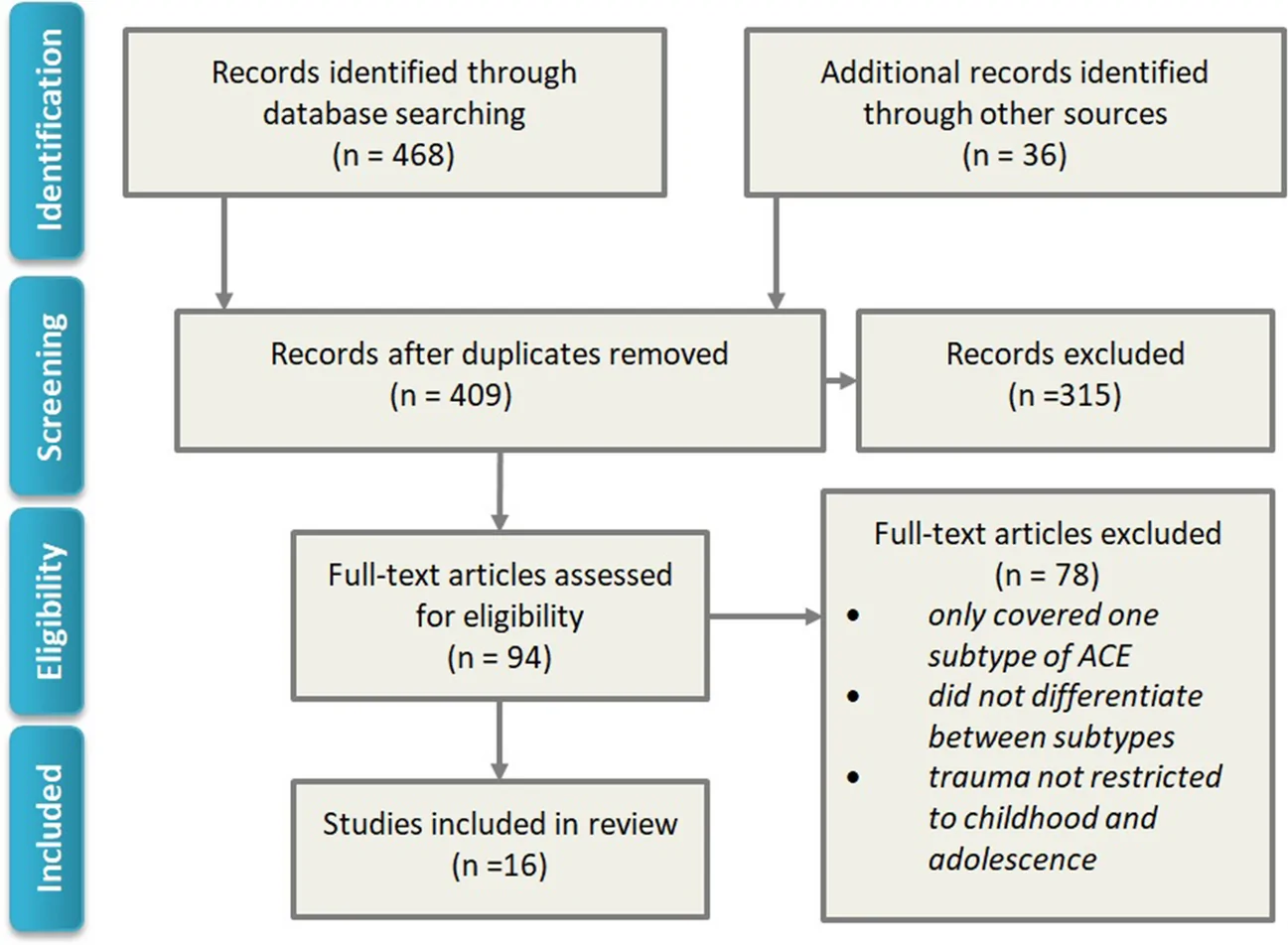
PRISMA flow diagram of search strategy

We conducted a comprehensive literature search of available electronic data bases (PubMed, MEDLINE, APA PsycInfo, Academic Search Ultimate) between 6–13 Sept. 2025 according to current Preferred Reporting Items for Systematic Reviews and Meta-Analyses (PRISMA) guidelines [68]. Searches included the following terms using Boolean operators: (RMD OR premenstrual symptoms OR premenstrual dysphoric disorder OR premenstrual syndrome OR premenstrual mood disorder OR PMS OR PMDD OR perimenopausal depression OR perimenopausal MDD OR perimenopause depression OR perimenopause MDD OR menopaus* depression OR menopaus* MDD) AND (adverse childhood experience OR childhood trauma OR childhood adversity OR early life stress OR child* abuse OR child* neglect OR ACE OR CTQ OR ELS). In addition to these databases, reference mining, citation chaining and searches in relevant journals generated further records (see Fig. 1). After duplicate removal, a pre-selection was made on the basis of title and abstract. Abstracts of the publications potentially to be included and the studies that could not be clearly assessed after a preliminary abstract review were included in the full-text analysis, 315 studies were excluded as outside of scope and not relevant. Assessment of records was conducted by two researchers and any discrepancies could be resolved by consensus. All remaining studies were retrieved in full text and checked for eligibility using defined inclusion and exclusion criteria developed for this purpose. This review included studies that (a) investigated associations between ACE (and related concepts) and RMDs, and (b) differentiated between subtypes of ACE. We included data from patient, university and community populations. Studies that investigated lifetime trauma without specific differentiation for age at trauma were excluded. One study [4] was excluded for quality reasons (insufficient description of methods and results). A final selection of 13 studies on menstrually related mood disorders and three studies on perimenopausal depression met our search criteria. For an overview of included studies, see Table 1.

**Table 1:** Summary of reviewed literature, * age of enrollment, ** descriptive study, *** in modified form

	Author	Year	Country	Population	Sample size	Age (mean/SD)	Study design	Statistical analysis	Diagnostic instrument	Trauma assessment
PMS & PMDD	Soydas et al	2014	Tükiye	patients	148	30,7 (6,0)	cross-sectional	ANOVA, correlation	PMSS	CTQ
	Bertone-Johnson et al	2014	USA	cohort	3.295	34,2 (4,1)*	longitudinal	logistic regression	COPE	CTQ***
	Azoulay et al	2020	Israel	students	112	23,4 (3,6)	cross-sectional	correlation	PSST	CTQ
	Wakatsuki et al	2020	Japan	community	204	32,4 (6,8)	cross-sectional	multiple regression, SEM	PMDD scale	CATS
	Gümüşsoy et al	2021	Tükiye	students	702	18,4 (1,1)	cross-sectional	correlation	PMSS	CTQ
	Ito et al	2021	Japan	patients	3815	35,1 (10,3)	cross-sectional	logistic regression	ACOG criteria	questions based on previous studies
	Younes et al	2021	Lebanon	students	318	21,8 (4,8)	cross-sectional	SEM	PSST	CASRS
	Yang et al	2022	Iceland	cohort	11,973	34,0 (9,1)*	longitudinal	Poisson regression	PSST	ACE-IQ***
	Kulkarni et al	2022	Australia	patients	100	36,5 (8,1)	cross-sectional	none**	C-PASS	WHO definition
	Kfoury et al	2024	Lebanon	students	915	27,1 (9,3)	cross-sectional	mediation analysis	PSST	CASRS-12
	Standeven et al	2024	USA	cohort	217	32,7 (4,4)	cross-sectional	logistic regression	PSST	CTE-S
	Kanamori et al	2025	Japan	community	2000	33,7 (6,4)	cross-sectional	logistic regression	MDQ, PMDD scale	ACE-Q***
	Saglam et al	2025	Tükiye	community	437	26,4 (5,8)	cross-sectional	t-tests, multiple regression	PMSS	CTQ
Perimenopausal	Epperson et al	2017	USA	cohort	243	41.6 (3.5)*	longitudinal	logistic regression	SCID	ACE-Q
depression	Shea et al	2021	Canada	patients	98	52.2 (4.8)	cross-sectional	General linear model	CES-D	CTQ
	Bromberger et al	2022	USA	community	333	45.7 (2.5)*	longitudinal	logistic regression	SCID	CTQ

Abbr: *SEM* Structural equation modeling, *PMSS* Premenstrual syndrome scale, *COPE* Calendar of premenstrual experiences, *PSST* Premenstrual symptoms screening tool, *ACOG* American college of obstetrics and gynecology, *C-PASS* Caroline premenstrual assessment scoring system, *MDQ* Menstrual distress questionnaire, *SCID* Structured clinical interview for DSM, *CES-D* Center for epidemiological studies – depression, *ACE-Q* Adverse childhood experiences questionnaire, *CTQ* Childhood trauma questionnaire, *CATS* Child abuse and trauma scale, *CASRS* Child abuse self-report scale, *CTE-S* Childhood traumatic event scale

## Review

### Menstrually related mood disorder

Several interesting and well-conducted studies were not included by us if they either did not differentiate between experiences in childhood/adolescence and trauma in adulthood in the statistical analyses [21, 31, 63] or studies that examined ACE exclusively as an aggregate variable and did not differentiate subtypes [61, 65, 67]. Of all the aforementioned disorder categories, the literature on PMS and PMDD was the most comprehensive. For the purpose of clarity, we have divided the presentation thematically into findings on the prevalence of individual ACE subtypes within the PMS/PMDD population, findings on the risk or odds for PMS/PMDD after exposure to these subtypes and associations between the severity of exposure to ACE subtypes and premenstrual symptoms. For an overview of the different instruments employed in these studies, see Table 2, for effects of ACE subtypes on these disorders, see Table 3.

**Table 2:** Overview of the instruments used in the studies under review

Adverse Childhood Experiences (ACE)		
ACE-(I)Q	Adverse Childhood Experiences—(International) Questionnaire	The ACE-Q is a self-report questionnaire on three dimensions: abuse, neglect, and household/family dysfunction—that are further divided into the subcategories physical, sexual, and emotional abuse; emotional and physical neglect; parental separation; household violence; parental substance abuse or psychiatric disorders; and household member in prison, 10 items with ten-point Likert-type scales. The ACE-IQ includes the further dimension of community/social adversity with the subcategories bullying, community violence and collective violence with a total of 29 items to recognize sources of childhood adversity outside the family context, age range: < 18y
CASRS	Child Abuse Self-Report Scale	Self-report questionnaire on four dimensions: psychological (14 items), neglect (11 items), physical (8 items) and sexual (5 items) on four-point Likert-type scale ranging 0 = Never to 3 = Always, a 12-item short version (CASRS-12) is available, psychometric validation literature is centered on Arab-speaking populations, age range: "childhood and adolescence"
CATS	Child Abuse and Trauma Scale	Self-report questionnaire on three dimensions, with a 14-item negative home atmosphere/neglect subscale, a 6-item sexual abuse subscale, and a 6-item punishment subscale, age range: "general atmosphere of your home when you were a child or teenager"
CTQ	Child Trauma Questionnaire	Self-report questionnaire on five dimensions, includes 70 items (short form includes 28 items): physical abuse, emotional abuse, sexual abuse, physical neglect, and emotional neglect, age range: < 18y
CTS-E	Childhood Traumatic Event Scale	Self-report questionnaire on six dimensions: death of a close friend or family member, major upheaval between parents (divorce, separation, etc.), traumatic sexual experience, physical violence, on seven-point Likert-type scales, age range: < 17y
Premenstrual Symptoms		
ACOG	American College of Obstetrics and Gynecology	Ito et al. [42] followed the ACOG definition and asked participants about 12 questions about affective and physical/behavioral symptoms that lasted for 3 to 13 days before menstruation in the previous 3 menstrual cycles
COPE	Calendar of Premenstrual Experiences	Self-report questionnaire of 26 affective and physical/behavioral, premenstrual symptoms during the past year on a 4-point Likert scale, with a range from none (0) to severe (4)
C-PASS	Caroline Premenstrual Assessment Scoring System	Standardized scoring system for DSM-5 based PMDD diagnoses that relies on symptom ratings with the Daily Record of Severity of Problems over ≥ 2 month
MDQ	Menstrual Distress Questionnaire	Self-report questionnaire with 44 items and 8 subdimensions, responses rated on six-point Likert scale ranging from 1 (no reaction at all) to 6 (acute or partially disabling), scores classified into menstrual symptoms severity as ‘moderate and below’ (lower 50th percentile of the score distribution), ‘slightly strong’ (25th–50th percentile), ‘strong’ (10th–25th percentile), and ‘very strong’ (top 10th percentile)
PSST	Premenstrual Symptoms Screening Tool	Self-report questionnaire consists of two sections. Section A includes 12 items assessing PMDD symptom severity on a 4-point scale, and respondents reporting any symptoms complete Section B, which contains 5 items evaluating impairment in work, school, social activities, and relationships; PMDD is diagnosed when at least one mood symptom is rated severe, at least four Section A items are rated moderate or severe, and at least one Section B item is rated severe
PSST	Premenstrual Symptoms Screening Tool	Self-report questionnaire with 44 items and nine subdimensions, responses rated on a five-point Likert-type scale ranging from 1 to 5, based on symptoms in the week before menstruation, PMS when the score is > 50%
PSST	Premenstrual Symptoms Screening Tool	Self-report questionnaire about premenstrual symptoms such as irritability, anxiety, mood swings, fatigue and physical discomfort and their impact on daily life, with 19 questions answered on 4-point Likert scales, with a range from none (0) to severe (4)
Perimenopausal Depression		
CES-D	Center for Epidemiologic Studies—Depression	Self-report questionnaire for assessing depressive symptoms, especially in large-scale epidemiological studies, included 20 items rated on three-point Likert-type scale
SCID	Structured Clinical Interview for DSM	Semistructured interview for making DSM-5 diagnoses

**Table 3:** Overview of ACE subtype-specific effects, Effect size: Cohen’s d, risk-, odds ratios, r and standardized betas were converted to Cohen’s d, prevalence ratios are directly reported, in case of “ Other subtype”, only the subtype with the highest effect is reported

Between-groups comparison of subtypes						
	Sexual abuse	Physical abuse	Emotional abuse	Physical neglect	Emotional neglect	Other subtype
Soydas et al. (2014)	0.438	0.433	0.487	-	0.487	-
Gümüşsoy et al. (2021)	0.296	0.404	0.479	0.053	0.030	-
Kfoury et al. (2024)	0.186	0.261	0.400	0.163^1^	0.163^1^	-
Saglam et al. (2025)	0.308	0.356	0.411	0.072	0.415	-
Association between trauma subtype exposure and RMD						
	Sexual abuse	Physical abuse	Emotional abuse	Physical neglect	Emotional neglect	Other subtype
Bertone-Johnson et al. (2014)	0.053	0.259	0.259	-	-	-
Ito et al. (2021)	0.259	0.368	0.235	-	0.082	0.105^1^
Yang et al. (2022)	1.37^PR^	1.28^PR^	1.44^PR^	1.22^PR^	1.51^PR^	1.50 ^PR,2^
Kanamori et al. (2025)	0.212	0.393	0.459	0.105	−0.238	0.736^3^
Bromberger et al. (2022)	−0.083^4^	0.324^4^	0.289^4^	−0.028^4^	0.027^4^	-
Association between trauma scores and symptom severity						
	Sexual abuse	Physical abuse	Emotional abuse	Physical neglect	Emotional neglect	Other subtype
Soydas et al. (2014)	0.455	0.453	0.479	-	0.479	-
Azoulay et al. (2020)	0.501	0.136	0.293	0.3388	0.404	-
Gümüşsoy et al. (2021)	0.364	0.464	0.624	0.140	0.082	-
Saglam et al. (2025)	0.240	0.347	0.285	0.201	0.436	-
Kanamori et al. (2025)	0.178	−0.181	0.123	0.414	−0.0283	0.525^5^

PR: prevalence ratio, *: study did not differentiate forms of neglect, 1: witnessing intimate-partner violence, 2: mental illness of a household member, 3: natural disaster, 4: without lifetime MDD at baseline, 5: parental mental illness

#### Are Certain ACE Subtypes More Frequent in Patients with PMS/PMDD?

In an early study [88], female outpatients with a PMDD diagnosis according to DSM-IV criteria, were compared in a between-groups design with healthy control participants. In addition to other variables, ACE was assessed using the CTQ. The group with PMDD had significantly higher CTQ total scores (*p* = 0.002), which were further broken down by subtype. There were significant differences between the groups for sexual (*p* = 0.012) and physical abuse (*p* = 0.009), as well as for emotional abuse and neglect (grouped together, *p* = 0.004), with higher scores in the PMDD group in all subscales. The dimension of physical neglect was not reported, although it is included in the standard CTQ assessment. However, the authors do not address this issue. In a further study, a larger but non-clinical sample was included [34]. This study recruited 702 female students and divided the sample according to PMS presence (PMS: *n* = 372). Screening for PMS was conducted with the Premenstrual Syndrome Scale (PMSS; [30] and ACE was assessed with the CTQ. Both groups were analyzed for the presence of ACE by comparing CTQ total and subscores using t-tests. The PMS group had significantly higher total CTQ scores and higher scores for emotional, sexual and physical abuse (all *p* < 0.001). However, the groups did not differ with respect to emotional neglect (p = 0.696) and physical neglect (*p* = 0.482). In a simple descriptive approach, [51] reported the frequencies of individual ACE subtypes in a sample of 100 women diagnosed with PMDD, based on a modified clinical assessment using the central 4 items of the Caroline Premenstrual Assessment Scoring System (C-PASS; [20], and compared those to the prevalence in the general female population. This study found PMDD patients to be more exposed to all forms of abuse and neglect, however, the difference to the general population was most pronounced for emotional abuse, which was experienced by 71% of the patients (compared to 9.1% in the reference population). Emotional abuse stood out as the by far most frequent form of ACE, with prevalence 46–50% higher than that of other subtypes: sexual (24%) and physical abuse (21%) and neglect (25%, reported as single entity). Although the comparison was descriptive and no statistical inferences can be drawn, the wide distribution of self-reported emotional abuse in this clinical population corresponds to the findings described above. In a study [46], the influence of ACE on PMS was investigated in an even larger sample (*n* = 915). Participants were recruited from the general public. The Premenstrual Symptoms Screening Tool (PSST; [90] was used to record PMS, while the 12-item short version of the Child Abuse Self Report Scale (CASRS-12; [46] was used to record ACE, with the four dimensions of psychological, physical and sexual abuse as well as neglect. The sample was divided on the basis of PSST scores according to the presence (*n* = 543) or absence of PMS and compared using t-tests for total and subscores of the CASRS-12. The PMS group showed significantly higher trauma exposure across all dimensions: participants with PMS were more affected by sexual abuse (*p* = 0.005), physical abuse (*p* < 0.001), psychological abuse (*p* < 0.001) and neglect (*p* = 0.016). A very recent study recruited 437 adult women with regular menstrual cycles and without psychiatric diagnoses by public announcements and screened for PMS symptoms using the PMSS [76]. The sample was divided into women with/without PMS (PMS *n* = 243) and compared using the independent groups t-test with regard to the ACE recorded. As above, ACE was recorded using the CTQ; all subscales were reported separately. There were highly significant group differences (*p* < 0.001) with regard to the overall score as well as for all forms of abuse and emotional neglect; women with PMS symptoms reported higher values. Only the physical neglect scale showed no group differences (*p* = 0.457), which could potentially also reflect the psychometric properties of this subscale. Taken together, PMS/PMDD affected women reported forms of abuse more frequently than those not affected by these conditions, especially emotional abuse.

#### Does The Risk or Odds of Developing PMS/PMDD Depend on ACE Subtype?

In an early study, [10] investigated the relationship between abuse in childhood or adolescence and PMS as part of a large prospective cohort study. The authors assessed the risk of developing moderate-to-severe PMS over a period of 14 years based on self-reported abuse, assessed with the Calendar of Premenstrual Experiences (COPE; [62]. The assessment of childhood and adolescent exposures to abuse was conducted with the CTQ, physical abuse in addition with the Revised Conflict Tactics Scale and sexual abuse with the Sexual Experience Survey. From these scales the authors created summary variables, based on an factor-analytical approach by [13], to capture the exposure to emotional, physical and sexual abuse in childhood. Even after adjustment for confounding factors, all three forms of abuse increased the risk for developing PMS, with stronger associations for physical abuse and emotional abuse than for sexual abuse. While women who reported of being exposed to emotional abuse during childhood were 2.6 times more likely to develop PMS, and had a 2.1-fold risk increase with physical abuse, sexual abuse was not consistently associated with risk of PMS. Of note, these associations did not substantially change after accounting for social support during this developmental period. These findings were largely corroborated in a later study [42]. In this study, 3940 women at a clinic in Japan were screened for PMS symptoms using a questionnaire based on the criteria of the American College of Obstetrics and Gynecology. The final sample consisted of 1018 women who fulfilled PMS criteria and 2277 control participants. A questionnaire from earlier studies by the authors was used to record ACE and multiple logistic regression analyses were conducted and odds ratios calculated to determine the risk for PMS based on previous ACE exposure. In addition to the three sub-types of abuse that were differentiated in the previous study, the participants were asked about emotional neglect and about witnessing intimate-partner violence. Again, physical and emotional abuse significantly increased the risk for PMS, even after adjustment for several health-related behaviors, with odds ratios of 1.89 and 1.55, respectively. This was not the case for emotional neglect and witnessing intimate-partner violence and, as above, sexual abuse, all of which were not significantly associated with PMS. A different approach was chosen by [104]: in a population based study that included 11,973 women, the authors investigated the association between ACE and premenstrual disorders, notably PMS and PMDD. The sample was derived from a large prospective cohort study launched in 2018. The PSST was used to classify participants into PMS (*n* = 2501), PMDD (*n* = 734) or no premenstrual disorder. Participants were screened for ACE using the modified Icelandic version of the World Health Organization (WHO) ACE-International Questionnaire (ACE-IQ). A high number of participants (77%) reported to have suffered from at least one form of ACE. The authors calculated log-binomial regression models and reported estimated prevalence ratios (PRs) for individual subtypes of ACE. To assess whether the ACE-associated risk for PMS/PMDD was modified by psychiatric comorbidities, the authors performed stratification analyses by including an interaction term between ACEs and psychiatric comorbidities separately in the model, and estimated PRs in the presence and absence of psychiatric comorbidities. Additionally, to examine the independent association between each type of ACE and PMS/PMDD, they adjusted each analysis for the other subtypes of ACEs to account for the interrelatedness of different types of ACEs. After adjustment for all confounding covariates, individual types of ACEs were all positively associated with PMDs (PRs: 1.11 to 1.51), with the strongest association observed for emotional neglect and abuse (and ‘household dysfunction: mental illness’). After mutual adjustment for other types of ACEs, the associations were considerably weaker but remained significant for sexual abuse, emotional neglect, family violence, mental illness of a household member, and peer and collective violence (PRs: 0.91 to 1.32). In a different study, 391 participants were selected for secondary analysis as a part from a prospective cohort, and divided into three groups [89]. Based on the PSST, they were classified into a premenstrual disorder group (PMD/PMDD) with *n* = 43, to a healthy control group (*n* = 217) and also a psychiatric control group (*n* = 102), i.e. patients with none or mild premenstrual symptoms and either a current mood or anxiety disorder or medication for a lifetime mood or anxiety disorder. Furthermore, and as the only study in this review, a group with premenstrual exacerbation (PME) was included (*n* = 38), that had moderate to severe premenstrual symptoms but otherwise fulfilled the criteria described for the psychiatric controls. The Childhood Traumatic Events Scale (CTE-S; [69] was used to screen participants for ACE which were differentially investigated by subtype. Univariate differences in type of ACE were compared between groups with Fisher’s exact test. To adjust for relevant demographic covariates, a further multinomial logistic regression with trauma type as the outcome was conducted. While groups differed with regard to sexual abuse and the category of other trauma (i.e. major upheaval during childhood or adolescence other than abuse or neglect), after controlling for medication use, race, income, and education with multinomial logistic regression models, no significant group differences in experience of sexual abuse or other trauma remained (all *p* > 0.15). In a very recent study, a large community sample of 2000 female participants was examined [44]. These were screened using the PMDD scale [60] and divided into a PMDD group (*n* = 101) and a non-PMDD group (*n* = 1899). Binomial logistic regression was used to determine the risk of PMDD based on the presence of ACE. These were recorded using the Japanese version of the broader ACE questionnaire (ACE-J), which, in addition to abuse and neglect, also includes adversities such as peer victimization, household dysfunction and community violence. In addition to an increased risk due to emotional abuse (OR = 2.44) as reported in previous studies, the highest association was interestingly found with the subcategory natural disaster (OR = 3.89). To summarize across studies, physical and emotional abuse seem to be the subtypes of ACE that most strongly increases the risk for developing PMS/PMDD, which corresponds to the findings of the above section.

#### Are Scores For Specific Ace Subtypes Associated With Severity of Premenstrual Symptoms?

[88] conducted a correlation analysis to assess the relationship between PMSS scores and CTQ total score as well as sub-scores, with all correlation revealing significant results (all *p* < 0.001): physical abuse (r = 0.221), sexual abuse (r = 0.222) and emotional abuse and neglect (r = 0.233). Again, physical neglect was not reported. Similarly, [6] investigated 112 undergraduate students for premenstrual symptoms, 22 participants fulfilled criteria for PMS and 16 for PMDD. Symptoms were assessed with the PSST, and correlated with total and sub-scores of the CTQ. Significant correlations were found for sexual abuse (r = 0.243, *p* < 0.01) and emotional neglect (r = 0.198, *p* < 0.05), not for the other forms of abuse or neglect. Yet, the authors also conducted a mediation path analysis and assessed direct and indirect effects for all subtypes, with emotion regulation as a mediator. When considering these indirect effects, all forms of abuse had a significant effect (*p* < 0.001) on PSST scores: emotional (R^2^ = 0.35), physical (R^2^ = 0.35) and sexual abuse (R^2^ = 0.36). Another study [96] investigated the influence of ACE on PMS in a nonclinical sample (*N* = 204). PMS were measured with the PMDD scale and ACE with the Child Abuse and Trauma Scale (CATS; [77], an instrument with the three subscales neglect or negative home atmosphere, punishment and sexual abuse. In this study, only neglect, which was not differentiated between emotional and physical forms, was positively correlated with symptom severity (*p* < 0.01). The study described above did not only compare the presence of ACE subtypes between groups with and without PMS [34], but also reported correlations between premenstrual symptoms and CTQ total and sub-scores. Positive correlations were found for the total score as well as all abuse sub-scores (all *p* < 0.001): emotional (r = 0.298), physical (r = 0.226) and sexual abuse (r = 0.179). Scores for emotional (r = 0.041, *p* = 0.278) and physical neglect (r = 0.07, *p* = 0.065) did not correlate with PMSS scores. A study [105] used structural equation modeling to investigate the relationship between ACE and PMS in a student sample (*n* = 318). PMS was assessed with the PSST, ACE was measured with the Child Abuse Self Report Scale (CASRS; [54], a 38 items inventory that can be divided into four categories: psychological, physical and sexual abuse as well as neglect. The model that was calculated also included depression, assessed with the Lebanese depression inventory (LDS-19), as a mediator. Sexual abuse (*p* = 0.02) and psychological abuse (*p* = 0.04), which correspond with the emotional abuse dimension of the CTQ, were positively related to symptom severity, yet this relationship was indirect and mediated by depression scores which again were strongly associated with PSST scores. Physical abuse (*p* = 0.84) and neglect (*p* = 0.96) were not associated with PSST scores. The above-mentioned study by [46], also investigated the role of an intermediary variable in the form of suicidal ideation in a mediation model. Thus, the hypothesis was tested that ACE leads to more suicidal ideation, which in turn affects PMS severity. This indirect mediation was shown for all subtypes of sexual abuse, physical abuse, psychological abuse and neglect at the *p* < 0.05 level. In addition, however, direct effects, which were independent of suicidal ideation, were also shown for the dimensions of physical (*p* = 0.024) and psychological abuse (*p* < 0.001) as well as neglect (*p* = 0.007), but not for sexual abuse (*p* = 0.198). In addition to the prevalence of CTQ subtypes in women with PMS (described above), the study by [76] used regression analysis to investigate whether certain subtypes of ACE are associated with increased levels of premenstrual syndrome in women. A significant influence was only found for emotional neglect (*p* = 0.006) and physical abuse (*p* = 0.039), but not for emotional (*p* = 0.206) and sexual abuse (*p* = 0.163) or physical neglect (*p* = 0.302). Finally, the study by [44] also investigated the association between scores for individual ACEs and symptom severity using the Menstrual Distress Questionnaire (MDQ). Ordinal logistic regression models were calculated, which, after adjustment for confounding variables, revealed that the subcategories of parental mental illness, physical neglect, childhood poverty, overcontrol, bullying, and sexual abuse were associated with premenstrual symptoms. The categories of emotional abuse and neglect, previously identified as risk factors, were not significantly associated; the biggest impact was found for parental mental illness (OR = 2.28). In summary, the associations between symptom severity and ACE seem to be strongest for emotional and sexual abuse.

### Perimenopausal Depression

Compared to the premenstrual disorders, we found considerably fewer studies for depression during the menopausal transition. As above, we did not include studies that did not differentiate between ACE subtypes [45, 59], and only three studies could be included in this review. Two of these only reported the percentages of subtypes in their sample; only one study compared them statistically between groups. Overall, there is less evidence for subtype-specific effects in this disorder.

One study investigated the influence of ACE the risk for a first episode of major depressive disorder during the menopausal transition [23]. The study was based on a subsample from a 16-year, longitudinal cohort study (Penn Ovarian Aging Study). Participants (*n* = 243) were midlife women (35–47 y) with the menopausal status assessed on the basis of menstrual bleeding data recorded using diaries, the number of menstrual periods between examinations and cycle length according to the stage system for reproductive ageing in women. Incident menopause depression was defined as new-onset MDD after the first occurrence of a ≥ 7-day change in cycle length, without a history of MDD, when menstrual cycles were regular. The assessment of ACE was based on the ACE-Q. The sample was divided in a low ACE group (0 or 1) and a high ACE group with a cutoff of ≥ 2 ACEs. The study found that women with two or more ACEs were more than twice as likely as those without or low ACE exposure to experience a first depressive episode during menopause transition. However, this analysis was not stratified for subtypes of ACEs, the authors only reported the number of ACEs by subtypes, with the most often reported subtypes being emotional abuse (23.5%), parental separation or divorce (22.2%) and living with someone who uses alcohol or drugs (21.8%), the least frequent forms were physical neglect (9.5%) and imprisonment of a household member (6.2%). Similarly, [86] addressed the question of whether depression in midlife women would be associated with a history of childhood maltreatment. This study recruited 96 women (40–65 y) seeking care at a clinic for bothersome menopausal symptoms. The time since the final menstrual period was used to classify women for menopausal status. Symptoms of depression were assessed by self-report with the Center for Epidemiological Studies—Depression questionnaire (CES-D; [71], menopausal symptoms (somatic symptoms, vasomotor symptoms, sexual function, anxiety and depression) with the Greene Climacteric Scale (GCS) and ACE with the CTQ. The impact of CTQ scores was analyzed with a general linear model, the study found that greater total CTQ scores were significantly associated with elevated depression scores and more severe menopausal symptoms. As in the previous study, the authors reported percentages for subtypes. The rate of ACE was higher than in [23] across subtypes (with different instruments employed for ACE), the most common type of abuse was emotional abuse (42.2%), followed by physical neglect (40.2%), emotional neglect (33.7%), physical abuse (20.6%) and sexual abuse (10.9%). Again, these subscores were not used in their analyses and can only be used for descriptive comparison to a reference population. The only study that investigated the contribution of ACE subtypes to the risk for depression during the menopause transition was [15]. Again, this analysis was part of a larger US-American cohort study (Study of Women’s Health across the Nation), in which participants were interviewed annually over a period of 15 years. The sample of 333 women (42–52 y) was classified by menopausal status based on menstrual bleeding patterns following WHO recommendations, with premenopause defined as no change in the regularity of menstrual bleeding, perimenopause as menstruation in the previous 3 months with an increase in bleeding irregularity or no menstruation in the previous 3–11 months and postmenopause was defined as the absence of menstruation for at least 12 months (incl. hysterectomy or bilateral salpingectomy). Depression was assessed by SCID-interviews. In contrast to [23], this study also included women with previous depressive episodes and also statistically compared women with lifetime depression and those with incident depression. Assessment of ACE was conducted with the CTQ, and repeated measures logistic regression models were calculated to determine the odds of developing a depressive episode during menopausal transition depending on ACE (and current stressors). These analyses were also stratified by participants with and without lifetime MDD at baseline. Significant interactions for emotional abuse (*p* = 0.022), emotional neglect (*p* = 0.002) and physical neglect (*p* = 0.038) were found for women with lifetime MDD, with odds for a depressive episode more than doubled for postmenopausal women affected by these ACE subtypes. In summary, it does not appear that certain subtypes would have a distinct role for this disorder.

### Perinatal and Postpartum Mood Disorder

As stated above, since an excellent meta-analysis on the topic has been published recently [26], reviewing individual studies at this point would contribute little to the literature. This analysis included 24 studies on the relationship between ACE and postpartum depression (PPD). A random effects model was calculated to pool 73 independent effects, and ACE was found to be a risk factor for PPD with an odds ratio (OR) of 2.31. In addition to this overall effect of ACE, the study also conducted subgroup analyses for individual subtypes. The strongest associations were found for emotional abuse (OR = 2.95, 95% CI [2.08, 4.20]) and emotional neglect (OR = 2.87, 95% CI [1.89, 4.36]), followed by sexual abuse (OR = 2.81, 95% CI [1.93, 4.09]). Other significantly associated subtypes of ACE were incarceration of family members (OR = 2.62, 95% CI [1.51, 4.54]), physical abuse (OR = 2.31, 95% CI [1.67, 3.19]) and physical neglect (OR = 2.15, 95% CI [1.36, 3.39]).

## Summary and Conclusion

The aim of this article was to provide an overview of available literature to address the question of subtypes-specific ACE associations with RMDs. Are certain ACE subtypes more common in women affected by RMDs when compared to other mental disorders? Is exposure to specific subtypes more strongly associated with the risk of these disorders or the severity of the symptoms? These questions are part of the ongoing debate about common or specific effects of ACE subtypes and their role in the etiology of mental disorders [14]. As mentioned in the introduction, emotional abuse was found to have a particularly detrimental effect and cause a greater risk of MDD than other subtypes. It can be assumed that this form of abuse leads to persistent and dysfunctional self-perceptions and low self-esteem and that such psychological processing patterns have an important mediating role [48]. However, it is still unclear whether this also applies to RMDs, with their close association to increased sex hormone sensitivity, or whether forms of abuse with more direct physical effects, such as sexual or physical abuse or neglect, do not have a greater impact.

We reviewed a number of studies with huge variation in terms of the population studied, the instruments used to assess ACE, study design and statistical methods. The sample size ranged from rather small (*n* < 100) to large (*n* > 10,000), some of the studies investigated patients with confirmed diagnoses, while most studies relied on student populations or recruited samples from the general population and used self-report measures for symptom assessment. Three articles covered subsamples of longitudinal cohort studies (although starting in adulthood and after ACE exposure), yet most studies used cross-sectional designs. ACE assessment was solely based on retrospective self-assessment, which is by its nature often the only available source of information. Most of the studies (seven out of sixteen) used the established CTQ, and we based our review on the subtypes corresponding with CTQ dimensions. Three further studies used the ACE-IQ (or versions translated or adapted into local languages), two studies used the CASRS and further studies used other instruments. Some instruments are much broader than the CTQ; the ACE-IQ includes 13 trauma categories, while others such as CATS are narrower and focus on three categories. Categories are grouped differently, for instance the CATS and CASRS do not differentiate between emotional and physical neglect, the CTE-S does not ask about emotional abuse or neglect. Although all instruments largely converge, there are differences in the psychometric properties, in the number and composition of the categories measured and in the sensitivity and specificity for individual subtypes, which opens the question of whether subtypes such as emotional or sexual abuse captures the same construct if measured with different instruments [84, 103]. Leaving the question of construct validity aside [27, 73], we found evidence for detrimental influences of all subtypes, with a diverse pattern of results between studies. Given the inherent problems of comparing and summarizing studies with differing levels of evidence, the negative influence seems to be highest for emotional abuse and lowest for physical neglect (see Table 3).

In all seven studies that reported the distribution of subtypes, emotional abuse was the most common. This in itself is no strong evidence, as this is also the most common subtype in the population as a whole [28, 102]. However, all studies that statistically compared subtype scores between groups showed a significantly higher proportion of the distribution than in the comparison group. Of the six studies that reported odds or prevalence ratios, four found significant associations for emotional abuse, which is more than for any other subtype and often with the highest ratios. These findings are consistent with the results of a recent review that summarized the influence of trauma (not limited to childhood) on PMDD, with emotional abuse most implicated in women with PMDD [32]. However, it is important to note that ACE subtypes usually do not occur in isolation, many participants report more than one form of trauma, and ACE subtypes often form larger clusters [80]. With emotional abuse being the most common subtype, it is often experienced in addition to other forms of ACE. With regard to the association between the ACE scores for the individual subtypes and the severity of the symptoms, the pattern is less clear; the number and the strength of the associations found suggest that sexual and emotional abuse as well as emotional neglect have comparable detrimental effects. Even if the influence of emotional abuse must be qualified with regard to the methodological approach, it can be clearly stated that emotional abuse and neglect as so-called silent forms of ACE are among the most detrimental subtypes of ACE and comparable, if not stronger, in their adverse effects.

How could a relationship between emotionally disruptive developmental conditions and hormonal processes be conceptualized? For the disorders in focus here, we hypothesized an increased sensitivity to fluctuations in sex hormones as a potential mechanism. These fluctuations are physiological and are associated with subclinical mood swings, irritability and other symptoms in many women. It can be assumed that women affected by the above-mentioned disorders are less able to compensate for them. [93] suggest that emotion dysregulation is a key mediating factor that interacts with physiological development of the gonadal hormone system. Again, this pathway would also correspond with the finding that emotional abuse plays such an important role in the etiology of these disorders. In their study, Epperson et al 23 differentiated between prepubertal ACE influences and those between the onset puberty and the age of 18 (postpuberty). Of note, this study found an increased risk of incident menopausal depression in women with postpubertal ACE exposure, with emotional abuse as the most common subtype in affected women, while prepubertal ACE was associated with a reduced risk. The authors discuss the postpubertal impact with reference to fluctuating estradiol during this developmental period which again has the potential to alter neurochemistry and brain structure and function. While the authors were also intrigued by the seeming capacity for prepubertal ACE exposure to promote resilience, even in the context of postpubertal ACE, they suggest a model of stress inoculation to explain this finding. Since these findings stand to be replicated and should be interpreted with caution, they highlight puberty as a particularly sensitive period for ACE influences. With the developmental stages of adrenarche and menarche, the incipient maturation of the gonadal system and the establishment of ovarian cyclicity are susceptible processes for disruptive stress effects. Interactive effects between hypothalamic–pituitary–gonadal (HPG) and hypothalamic–pituitary–adrenal (HPA) axis have been described [3, 37] and might contribute to what has been described as pubertal stress recalibration [70]. [36] proposed a mechanism how ACE may increase PMDD vulnerability through altered GABA-A receptor sensitivity and HPA-axis dysregulation: chronic stress exposure during sensitive developmental periods would affect the stress-sensitive GABAergic neuroactive steroid system and thereby alter GABA-A receptors and neurosteroid signaling, leaving affected individuals susceptible to cycle-related progesterone fluctuations in later life. Emotional abuse and neglect might therefore contribute to the complex interplay between emotional dysregulation and dysregulation of the HPG and HPA axis.

We reviewed the literature to address the question whether individual subtypes are more implicated in RMDs than others. A follow-up question that cannot be addressed with the findings published so far is whether different subtypes are perhaps mediated by different mechanisms. An indication of this comes from a study that assessed hormonal and mood data across the menstrual cycle (for one month) in patients with PMDD, classified according to lifetime sexual abuse, physical abuse or both [21]. This study interestingly found differential effects of physical and sexual abuse on hormonal patterns of symptom reactivity: those with a history of physical abuse had a closer association between progesterone fluctuations and mood or interpersonal symptoms, while those with a history of sexual abuse reported more pronounced estradiol-dependent anxiety symptoms (compared to those without abuse). The authors interpret the stronger association between physical abuse and progesterone with reference to an increased salience of social stimuli following a rising level of this hormone. While this increased attention to social cues would lead to pleasant emotions and signal opportunities in individuals with a history of positive interpersonal experiences, this might lead to greater interpersonal sensitivity and expectations of harm and hostility in women exposed to physical abuse. Following the authors, negative social cognitions and expectations would be stronger in physical abuse since this subtype, compared to sexual abuse, is more often perpetrated by a biologically-related, central attachment figure with resulting associations between cyclical changes in social salience and cognitive expectations of harm. In contrast, cyclic elevations of estradiol were more strongly associated with anxiety symptoms in women with a history of sexual abuse. The authors attempt to explain this link by the fact that estradiol coincides with a peak maximum at ovulation and thus with peak fertility and is associated with increased sexual interest and desire. This increased orientation towards sexual cues could in turn activate abuse-related symptoms and trigger anxiety in affected women. Yet, this study only contrasted physical and sexual abuse, the potential impact and mediating mechanisms of emotional abuse or neglect were not addressed. In a further study, [75] investigated the moderating effect of changes in estradiol levels on trauma-related symptoms in female PTSD patients. This study conducted ecological momentary assessment and hormone samples across the cycle and also considered trauma characteristics such as type, chronicity, and timing. An interesting pattern emerged, with an association between lower estradiol levels and higher severity of symptoms but only in women who suffered from sexual trauma. Compared to nonsexual trauma, women who were affected by sexual trauma reported increased symptom severity in low-estradiol states and similar symptom severity in high-estradiol states. While this finding could not be replicated in a very recent study, indirect effects of progesterone could be demonstrated [11]. Here, no interactive effect between trauma and endogenous estradiol on PTSD symptoms was found. A regression analysis revealed that progesterone effects were moderated by age at trauma: progesterone was negatively associated zeilenuwith symptom severity if the trauma occurred in childhood or adolescence and positively associated if the trauma occurred in adulthood. Although these individual findings need to be confirmed by further studies, it highlights potential biological pathways that might differ depending on type of trauma and age of exposure. Another outstanding research question is how adult trauma exposure and stressful life events relate to the effects described here. Some of the studies discussed here assessed these variables and included them in the statistical models [10, 15, 46, 96, 105]. Across these studies, the overall pattern suggests that ACE remain associated with RMD outcomes even after accounting for adult stressor. While evidence for formal mediation is limited, these stressful events in later life contribute an additional independent risk. Ultimately, the question of a genetic predisposition to hormonal sensitivity and its relationship to ACE would need to be further investigated. Such a predisposition could possibly be inherited from mother to daughter, while its genetic expression would be moderated by environmental influences such as ACE. However, this environment is a social environment, which in most subtypes of ACE is largely shaped by the parents. Even though the existing literature already reveals considerable information about the association between ACE subtypes and RMDs, many open questions remain to be answered. Subtype-specific mechanisms of effect mediation, predisposing factors that increased the risk for certain disorders after ACE exposure and whether certain subtypes of ACE have particularly harmful effects at certain ages (i.e. pre- vs. postpubertal) will hopefully be addressed in future research programs. Finally, this review should conclude on a hopeful note. Even though we have presented evidence from many sources pointing to a predisposing effect, such experiences do not necessarily lead to later psychiatric disorders. As described earlier, stressors later in life contribute independently to the risk, which provides a lever for preventive interventions. Across many studies, social support emerged as the most important resilience factor in buffering ACE [16, 53], and in some of the studies included here, specifically for premenstrual disorders [10, 104]. However, in cases of emotional abuse in particular, key family caregivers are not available to provide support. It is therefore a societal responsibility to ensure supportive relationships through schools, clubs, social workers, and the wider community.

## Key References

- Andersen, E. H., Nagpal, A., Eisenlohr-Moul, T. A., & Gordon, J. L. (2024). A novel method for quantifying affective sensitivity to endogenous ovarian hormones. Psychoneuroendocrinology, 167, 107,095. https://doi.org/10.1016/j.psyneuen.2024.107095
  - ○ This study describes a method for quantifying affective sensitivity to endogenous fluctuations in estradiol and progesterone using time-lagged cross-correlations between repeated endogenous hormone levels and self-reported affect.
- Fu, C., Li, C., Wan, X., Yang, Y., Zhang, S., & Hu, J. (2024). The Relationship Between Adverse Childhood Experiences and Postpartum Depression: A Systematic Review and Meta-Analysis. Trauma Violence Abuse, 25(4), 3066–3081. https://doi.org/10.1177/15248380241235639
  - ○ This review provides an overview of studies that investigate the influence of ACE on postpartum depression. As in our article, the correlations were differentiated according to ACE subtypes.
- Kundakovic, M., & Rocks, D. (2022). Sex hormone fluctuation and increased female risk for depression and anxiety disorders: From clinical evidence to molecular mechanisms. Front Neuroendocrinol, 66, 101,010. https://doi.org/10.1016/j.yfrne.2022.101010
  - ○ This article examines the role of fluctuating sex hormones as a key biological risk factor for depression and anxiety in women through their influence on brain structure and function. The authors present chromatin organization as a molecular mechanism controlled by sex hormones that regulates neuronal gene expression and brain plasticity and might lead to psychopathologies through epigenetic influences.
- Tonon, A. C., Ramos-Lima, L. F., Kuhathasan, N., & Frey, B. N. (2024). Early Life Trauma, Emotion Dysregulation and Hormonal Sensitivity Across Female Reproductive Life Events. Curr Psychiatry Rep, 26(10), 530–542. 10.1007/s11920-024–01527-y
  - ○ This reference presents a theoretical model for the influence of neurobiological changes resulting from early trauma and consequently increased sensitivity to fluctuations in sex hormones in adulthood, which, in conjunction with emotional dysregulation, increases the risk of affective disorders.

## Data Availability

No datasets were generated or analysed during the current study.

## Footnotes

1 The conditions described in this article could also be experienced by individuals who might not identify as women. In this paper, we use the term women/females interchangeably and refer to individuals who have been assigned female sex at birth.
